# The association of health anxiety with low back pain and physical inactivity in Chinese university students: a structural equation modeling study

**DOI:** 10.3389/fpsyg.2025.1685025

**Published:** 2026-03-11

**Authors:** Songyang Lai, Xiaoyu Feng, Biaoqi Xu, Hamid Amini

**Affiliations:** 1 Department of Sports Training, Nanjing Sport Institute, Xuanwu District, Nanjing, Jiangsu, China; 2 Department of Physical Education, Nanjing University, Qixia District, Nanjing, Jiangsu Province, China; 3 Department of Physical Education, Guangdong University of Education, Haizhu District, Guangzhou, Guangdong, China; 4 Department of Physical Education, University of Tolo-e-Mehr, Qom, Iran

**Keywords:** health anxiety, low back pain, physical activity, physical education, sedentary behavior, universities

## Abstract

**Background:**

Health anxiety involves excessive worry about illness and heightened vigilance toward bodily sensations. These concerns may relate to reduced physical activity and musculoskeletal symptoms such as low back pain (LBP). However, the interrelationships among health anxiety, physical inactivity, and LBP in Chinese university students remain insufficiently explored. This study examined these associations using structural equation modeling (SEM).

**Methods:**

A cross-sectional survey was conducted between February and May 2025 among 5,607 third- and fourth-year students from 39 universities in Guangdong Province, China. Standardized instruments measured health anxiety, physical activity, participation in physical education (PE) classes, and LBP. SEM was used to evaluate direct and indirect associations while adjusting for demographic and behavioral covariates.

**Results:**

Among participants (64.99% female; 22.32 ± 0.67 years), 44% were classified as physically inactive and 33.93% reported LBP in the past month. Higher health anxiety was associated with a greater likelihood of physical inactivity (*β* = 0.291), and an indirect association with LBP through inactivity (indirect effect = 0.061). Physical inactivity was associated with greater LBP intensity (*β* = 0.211) and higher BMI (*β* = 0.157), and BMI was associated with higher LBP (*β* = 0.165). Participation in PE classes showed an inverse association with physical inactivity (*β* = −0.119), indicating that students who attended PE classes more frequently were less likely to be classified as inactive. Gender and family income showed negligible associations with health anxiety.

**Conclusion:**

Health anxiety was linked to both physical inactivity and LBP, and physical inactivity partially accounted for this association. The findings underscore the importance of integrated approaches addressing both psychological concerns and activity-related behaviors among university students. Longitudinal studies are needed to clarify temporal pathways and evaluate whether interventions targeting health anxiety or sedentary behavior may help reduce LBP in young adults.

## 1 Introduction

Health anxiety refers to an ongoing, excessive worry about having or developing a serious illness, often triggered by heightened attention to normal bodily sensations and misinterpretation of benign physiological changes as symptoms of disease (Lebel et al., 2020). This form of anxiety can lead to maladaptive behaviors such as frequent reassurance-seeking, avoidance of physical activity, and excessive monitoring of bodily states, patterns that may paradoxically intensify the anxiety itself (Luo et al., 2021). These responses not only reinforce anxiety but may also contribute to the development or persistence of physical symptoms, including chronic pain conditions (Arango-Dávila and Rincón-Hoyos, 2018).

Low back pain (LBP) is one of the most prevalent musculoskeletal conditions worldwide and a leading cause of disability, including among young adults (Hartvigsen et al., 2018; Ferreira et al., 2023). Although China reports relatively low age-standardized LBP prevalence rates, the absolute number of affected individuals remains high due to its large population (Xu et al., 2024). In most cases, LBP is non-specific and not attributable to identifiable pathological conditions; instead, it is associated with behavioral and psychosocial factors such as physical inactivity, elevated body mass index (BMI), and psychological distress (Ferreira et al., 2023).

Recent research has increasingly highlighted the role of psychological factors—especially health anxiety—in shaping the experience and reporting of musculoskeletal pain (Hnatešen et al., 2022; Zale and Ditre, 2015). Individuals with elevated health anxiety often exhibit heightened pain sensitivity, lower pain thresholds, and a tendency to catastrophize bodily sensations (Hnatešen et al., 2022; Zale and Ditre, 2015). Cognitive-behavioral mechanisms such as fear-avoidance beliefs and threat-oriented appraisal may discourage physical activity, promote muscle deconditioning, and amplify pain perception (Zale and Ditre, 2015; Baird and Sheffield, 2016). However, the complex interplay between psychological distress, behavioral avoidance, and somatic symptoms remains poorly understood, and cross-sectional designs limit causal inference.

Physical inactivity may function both as a consequence and a contributor to health anxiety and LBP. Among university students, fear of injury or symptom exacerbation may lead to avoidance of physical activity (Yalçınkaya et al., 2022; Landi et al., 2020), while regular physical activity has been shown to reduce anxiety through mechanisms such as endorphin release, improved emotion regulation, and reduced physiological reactivity to stress (Svensson et al., 2021; Wanjau et al., 2023; Gaudlitz et al., 2023). Despite these benefits, physical inactivity is widespread among Chinese university students, with approximately 75% failing to meet recommended physical activity guidelines (Wang et al., 2017). This inactivity increases vulnerability to obesity, psychological distress, and musculoskeletal conditions such as LBP (Lindell and Grimby-Ekman, 2022; Alzahrani et al., 2019; Bogdanis, 2012). Participation in physical education (PE) classes may also influence students’ activity habits and psychological resilience. However, declining PE requirements in Chinese universities have led to reduced engagement, and the potential protective role of PE participation remains understudied.

Existing studies have typically examined health anxiety, physical inactivity, and LBP separately, often relying on bivariate associations rather than modeling their interrelated behavioral and psychological mechanisms. Furthermore, previous findings regarding the associations among physical inactivity, BMI, and pain severity have been inconsistent across student and non-student populations, highlighting the need for analytic approaches capable of testing multiple pathways simultaneously. Cultural norms regarding help-seeking, pain expression, and academic pressure may also shape anxiety-related cognitions and physical activity patterns differently among Chinese students. Yet, limited research has examined how behavioral (e.g., inactivity), physiological (e.g., BMI), and psychological (e.g., health anxiety) correlates operate together within a unified framework, leaving uncertainty about their relative contributions and potential pathways. These gaps underscore the need for an integrated analytical approach to clarify how these factors co-occur within youth populations living in highly competitive academic environments.

To address these gaps, the present study investigates the associations between health anxiety, physical inactivity, and LBP among Chinese university students. Using structural equation modeling (SEM), we aim to evaluate both direct and indirect pathways linking psychological and behavioral factors with musculoskeletal outcomes. Findings may inform targeted interventions to promote physical and psychological well-being in university settings, addressing the dual burden of anxiety and pain among students.

## 2 Methods and materials

### 2.1 Participants and study design

This cross-sectional study was conducted between February and May 2025 across Guangdong Province, China—one of the most populous and socioeconomically diverse provinces in the country. This province-wide design enabled the inclusion of a broader range of institutional types and student backgrounds, enhancing the generalizability of findings. Guangdong hosts approximately 160 accredited universities and colleges offering undergraduate programs, including public universities, private institutions, and vocational colleges.

The study targeted third- and fourth-year undergraduate students. Upper-year students were selected to allow examination of behavioral and psychological patterns in more experienced students, as they may have greater familiarity with managing academic and personal responsibilities. This approach provides a practical framework to explore variations in health-related behaviors and musculoskeletal outcomes within a more developmentally mature cohort.

A stratified cluster random sampling approach was employed. Institutions were first stratified by type (public, private, vocational), and 39 universities were randomly selected in proportion to their representation within the province. Within each selected institution, one or more classes of third- or fourth-year students were randomly chosen, and all students present during data collection were invited to participate.

The required minimum sample size was estimated using G*Power 3.1, assuming a medium effect size (*f*^2^ = 0.15), *α* = 0.05, statistical power = 0.95, and five predictors (BMI, physical activity, participation in PE classes, health anxiety, and family income). This calculation indicated a minimum requirement of 1,038 participants. To account for an anticipated 15–20% nonresponse or attrition rate, the minimum target sample size was adjusted to approximately 1,246 participants.

Data were collected through supervised, in-class, paper-and-pencil questionnaires administered by trained research assistants. After obtaining institutional approval, assistants visited selected classes during scheduled hours, provided a standardized explanation of the study’s purpose and confidentiality measures, and distributed the surveys. Students completed the questionnaires anonymously and returned them immediately on site. This procedure minimized missing data and ensured consistency across institutions.

Eligible participants were full-time third- or fourth-year undergraduates aged 18–25 years who were able to complete the questionnaire independently. Students were excluded if they reported LBP due to known pathological conditions (e.g., spinal tumors, fractures), had recent sports-related injuries affecting the lower back, were majoring in physical education or sports sciences (due to atypically high baseline activity levels), or attended university fewer than 3 days per week, indicating irregular academic engagement.

All participants provided written informed consent. The study protocol was approved by the Research Ethics Committee of Guangdong General Hospital, Guangdong Academy of Medical Sciences (Code: 20241110-GDR), and all procedures complied with the ethical standards outlined in the Declaration of Helsinki.

### 2.2 Variables

For modeling, values were assigned to observed variables including gender, family annual income, BMI, physical inactivity, participation in physical education classes, health anxiety, and LBP (Table 1). All variables were treated as observed indicators in the structural equation model.

**Table 1:** Assignment of observed variables.

Variables	Value
1. Gender	1 = Male, 2 = Female
2. Family annual income (Yuan)	1 = <300 k; 2 = 300–500 k; 3 > 500 k.
3. BMI (kg/m^2^)	≥0, continuous variable
4. Physical inactivity	0 = active (≥600 MET.min/week), 1 = inactive (<600 MET.min/week)
5. Health anxiety	0–7, continuous variable
6. Participate in physical education classes (days/month) 0–8, continuous variable	
7. LBP intensity (VAS score) 0–10, continuous variable	

BMI, Body mass index; MET, Metabolic equivalent of task; LBP, Low back pain.

### 2.3 Assessments

#### 2.3.1 Sociodemographic and anthropometric variables

Gender was assessed via self-report. Family annual income was recorded using a four-level ordinal scale (<300 k, 300–500 k, and >500 k yuan), allowing adjustment for socioeconomic status. BMI was calculated using self-reported height and weight, applying the standard formula of weight (kg) divided by height in meters squared. Interpretation of BMI followed the classification system recommended by the National Health Commission of China, which is widely used in Chinese epidemiological research (Pan et al., 2025). According to these criteria, BMI values <24 kg/m^2^ indicate underweight or normal weight and values of ≥24.0 kg/m^2^ indicate overweight or obesity.

#### 2.3.2 Health anxiety

Health anxiety was measured using the Whiteley Index-7, a validated instrument consisting of seven dichotomous (yes/no) items. (Pilowsky, 1967). Total scores range from 0 to 7, with higher scores reflecting greater health-related worry. The Chinese version of the scale has demonstrated satisfactory internal consistency and construct validity in previous study (Lee et al., 2011). In the current study, the Cronbach’s *α* coefficient was 0.72, indicating acceptable reliability.

#### 2.3.3 LBP

In this study, LBP was defined as pain in the lower back not attributable to pathological conditions such as vertebral fractures or tumors. To aid comprehension, the questionnaire included a simple illustration of the lower back region. Participants were asked: “In the last month, have you experienced LBP on most days of the week (i.e., four or more days)?” If the response was affirmative, pain intensity was assessed using the Visual Analogue Scale (VAS), a 10-point scale ranging from 0 (no pain) to 10 (extreme pain), with higher scores indicating greater pain severity (Heller et al., 2016).

#### 2.3.4 Physical inactivity

Physical activity was measured using the short version of the International Physical Activity Questionnaire (IPAQ), which evaluates the frequency and duration of walking, moderate, and vigorous-intensity activities (Amini et al., 2020). Each activity is assigned a MET value—3.3 for walking, 4.0 for moderate, and 8.0 for vigorous intensity—representing multiples of the resting metabolic rate. Weekly MET-minutes were calculated by multiplying the MET value by the total minutes spent on each activity and summing across all categories. Participants with less than 600 MET-minutes per week were classified as physically inactive (Tran et al., 2020). The Chinese version of the IPAQ has demonstrated acceptable reliability in adult populations (*r* > 0.70) (Macfarlane et al., 2007), and in present study, the Cronbach’s *α* was 0.81, indicating good internal consistency.

#### 2.3.5 Participation in PE classes

In China, PE classes are compulsory during the first two undergraduate years but optional thereafter. For each participant, the number of PE classes attended in the past month was recorded. The intensity and content of PE classes were not assessed.

### 2.4 Statistical methods

All statistical analyses were performed using IBM SPSS Statistics 26.0 and AMOS 26.0 (IBM Corp., Armonk, NY, USA). Significance was set at *p* < 0.05 (two-tailed). Descriptive statistics (means ± standard deviations for continuous variables; frequencies and percentages for categorical variables) were calculated to describe characteristics of the participants. Independent-samples *t*-tests were performed to compare physical activity levels, BMI, LBP, and participation in physical education classes between students with and without health anxiety. Pearson’s correlation coefficients were used to examine bivariate relationships among study variables.

Structural equation modeling (SEM) was used to evaluate direct and indirect associations among health anxiety, physical inactivity, and LBP, while controlling for gender, family income, BMI, and participation in PE classes. The hypothesized model included physical inactivity as a mediator between health anxiety and LBP. Other variables were treated as observed variables. Parameters were estimated using maximum likelihood. To address potential deviations from multivariate normality and to obtain robust confidence intervals for indirect effects, bias-corrected bootstrapping with 2,000 resamples was applied. Model fit was evaluated using Chi-square test (*χ*^2^), Goodness-of-fit index (GFI), adjusted goodness-of-fit index (AGFI), Comparative fit index (CFI), Tucker–Lewis index (TLI), Normed fit index (NFI), and Root mean square error of approximation (RMSEA) (Byrne, 1994; Hu and Bentler, 1999; Schumacker and Lomax, 2004; Wei, 2014). Standardized estimates for direct, indirect, and total effects were reported along with 95% bias-corrected confidence intervals. The overall response rate was 94.15%. Item-level missingness was minimal; missing data were handled using full information maximum likelihood (FIML) under the assumption of missing at random (MAR). Residual covariances among conceptually related variables were freely estimated when theoretically justified to account for shared variance not captured by directional paths. Model modifications were guided by theoretical plausibility, and post-hoc data-driven changes without theoretical support were avoided.

## 3 Results

Of the 5,955 questionnaires distributed across the universities, 5,607 met inclusion criteria and were included in the analysis, corresponding to a valid response rate of 94.15%. The participants had a mean age of 22.32 ± 0.67 years, and 64.99% were female (*n* = 3,644).

**Figure 1:**
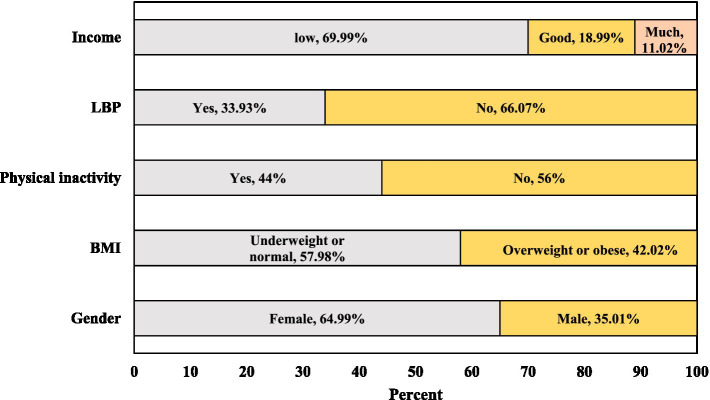
Participant characteristics, expressed as percentages; LBP, lower back pain; BMI, body mass index.

Regarding socioeconomic status, 69.99% (*n* = 3,924) of students reported a family annual income below 300,000 yuan, 18.99% (*n* = 1,065) between 300,000 and 500,000 yuan, and 11.02% (*n* = 618) above 500,000 yuan. In terms of BMI distribution, 57.98% (*n* = 3,251) were underweight or normal and 42.02% (*n* = 2,356) were overweight or obese. Participants accumulated an average of 871.41 ± 620.21 MET-minutes/week of physical activity. Based on the established cut-off of 600 MET-minutes/week, 44% (*n* = 2,467) were classified as physically inactive. Additionally, 33.93% (*n* = 1,906) reported experiencing LBP during the past month (Figure 1). The mean health anxiety score was 1.82 ± 0.89, and participation in physical education (PE) classes averaged 1.95 ± 1.26 days per month.

Pearson correlation coefficients are presented in Table 2. BMI was negatively associated with physical activity (*r* = −0.388, *p* < 0.01) and participation in PE classes (*r* = −0.266, *p* < 0.01), and positively associated with LBP intensity (*r* = 0.143, *p* < 0.01). Physical activity showed positive associations with participation in PE classes (*r* = 0.232, *p* < 0.01) and negative associations with both health anxiety (*r* = −0.164, *p* < 0.01) and LBP (*r* = −0.175, *p* < 0.01). Health anxiety demonstrated a small but significant positive association with LBP (*r* = 0.216, *p* < 0.01). Family income and gender showed negligible or non-significant correlations with the primary variables.

**Table 2:** Correlation matrix for the study variables.

Variable	Family annual income	BMI	Physical activity	Health anxiety	Participate in physical education classes	Back pain
Family annual income	1	0.02	0.091	0.083	0.012	−0.079
BMI	0.02	1	−0.388^**^	0.05	−0.266^**^	0.143^**^
Physical activity	0.091	−0.388^**^	1	−0.164^**^	0.232^**^	−0.175^**^
Health anxiety	0.083	0.05	−0.164^**^	1	−0.036	0.216^**^
Participate in physical education classes	0.012	−0.266^**^	0.232^**^	−0.036	1	0.084
Back pain	−0.079	0.143^**^	−0.175^**^	0.216^**^	0.084	1

^**^P < 0.01, ^*^P < 0.05; Pearson correlation was used to explore the correlation between variables; BMI = body mass index.

**Figure 2:**
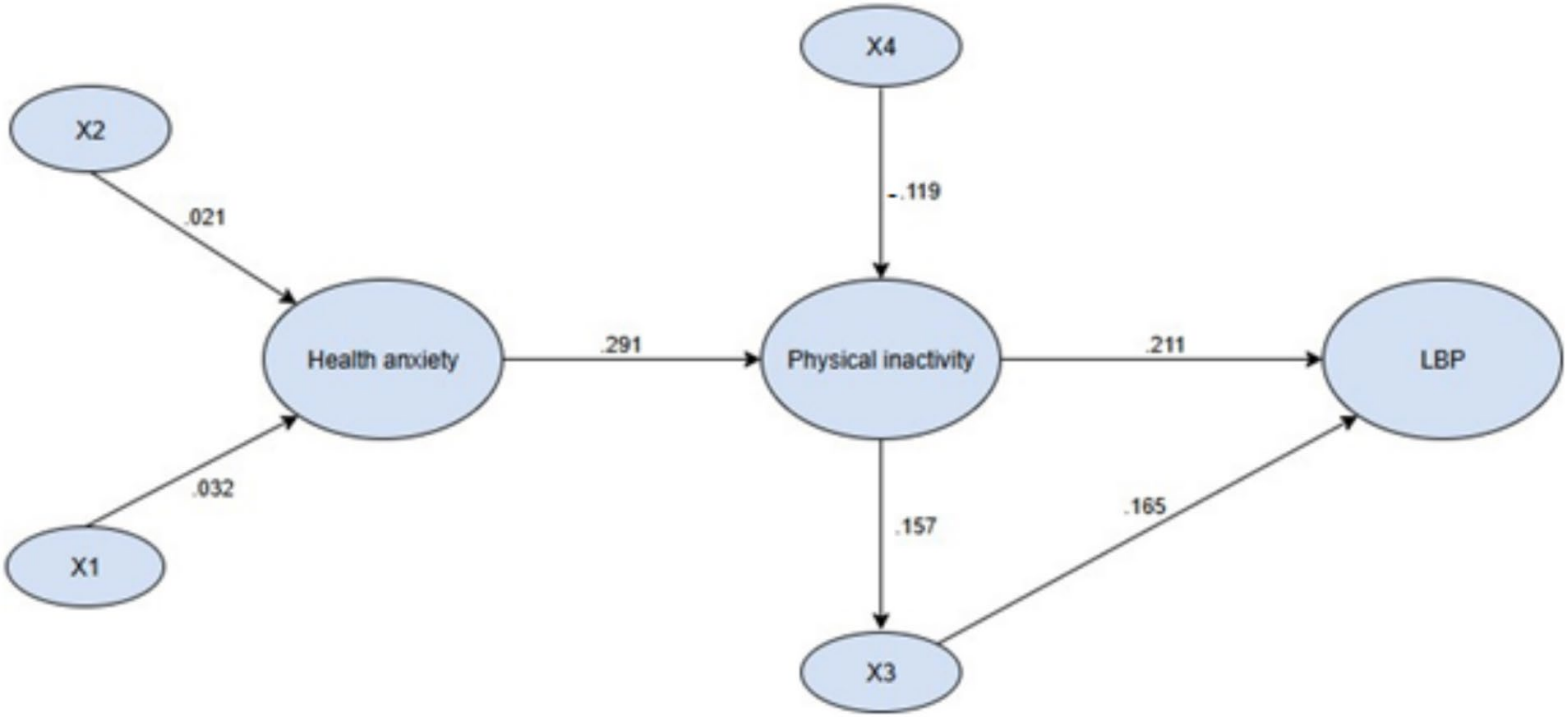
The final structural equation model; X1: gender; X2: family annual income; X3: BMI; X4: participation in physical education classes (*p* < 0.001).

A SEM was constructed to examine the relationships among health anxiety, physical inactivity, and LBP (Figure 2). The model demonstrated good fit to the data (*χ*^2^ = 422.021, *p* < 0.001; GFI = 0.962; AGFI = 0.951; RMSEA = 0.05).

A summary of standardized path coefficients is presented in Table 3. Health anxiety was positively associated with physical inactivity (*β* = 0.291, 95% CI [0.20–0.37]). Although cross-sectional data preclude causal inference, this association suggests that students reporting greater health-related concerns were more likely to fall into the physically inactive category. Health anxiety also demonstrated a significant indirect association with LBP through physical inactivity (indirect effect = 0.061, 95% CI [0.03–0.10]). Physical inactivity, in turn, was directly associated with higher LBP intensity (*β* = 0.211, 95% CI [0.14–0.28]). Additionally, physical inactivity showed a direct positive association with BMI (*β* = 0.157, 95% CI [0.08–0.21]). BMI also demonstrated a positive association with LBP intensity (*β* = 0.165, 95% CI [0.12–0.26]), resulting in a small but significant indirect pathway linking inactivity to LBP through BMI (*β* = 0.026, 95% CI [0.01–0.05]). Participation in physical education classes demonstrated a negative association with physical inactivity (*β* = −0.119, 95% CI [−0.18 to −0.06]). This coefficient indicates that students who attended PE classes more frequently were less likely to be classified as physically inactive. This pattern aligns with the expectation that greater engagement in structured physical activity opportunities is generally associated with higher overall activity levels.

**Table 3:** Standardized direct, indirect, and total effects with 95% bias-corrected confidence intervals.

Exogenous variable	Endogenous variable	Direct effect	Indirect effect	Total effect
Gender	Health anxiety	0.032 (0.01–0.06)		0.032
Family annual income	Health anxiety	0.021 (0.00–0.04)		0.021
Health anxiety	Physical inactivity	0.291 (0.20–0.37)^**^		0.291^**^
	LBP		0.061 (0.03–0.10)^**^	0.061^**^
Physical inactivity	LBP	0.211 (0.14–0.28)^**^	0.026 (0.01–0.05)^**^	0.237^**^
Physical inactivity	BMI	0.157 (0.08–0.21)^**^		0.157^**^
BMI	LBP	0.165 (0.12–0.26)^**^		0.165^**^
Participate in physical education classes	Physical inactivity	−0.119 (−0.18 to −0.06)^**^		0.119^**^

^**^P < 0.01; structural equation modeling was used to evaluate the relationships among the variables.

Gender and family income showed no statistically significant associations with health anxiety.

## 4 Discussion

Understanding how psychological concerns intersect with behavioral patterns is important for interpreting musculoskeletal symptoms among university students. In the present study, SEM was applied to evaluate how health anxiety, physical inactivity, PE participation, and BMI jointly relate to LBP.

The finding that students with higher health anxiety were more likely to be inactive may reflect heightened sensitivity to internal bodily cues, which can make physical engagement feel uncertain or uncomfortable. While this aligns with principles of avoidance-based models, the current data indicate that anxious students tended to adopt lower-activity lifestyles rather than clarifying the underlying mechanism (Kirmizi et al., 2021; Walsh et al., 2024). The present study also found that health anxiety was indirectly associated with LBP through physical inactivity. Although this pattern is consistent with theoretical models linking psychological distress and musculoskeletal symptoms, these relationships should be interpreted as associations rather than causal effects given the cross-sectional design (Yıldırım et al., 2019).

Avoidance of movement and activity, particularly in young adults, can plausibly impair core stability, reduce spinal flexibility, and lower pain tolerance thresholds, potentially increasing vulnerability to LBP (Skundric et al., 2021). In our SEM, physical inactivity exhibited a significant direct path to LBP as well as a significant direct path to BMI (i.e., standardized path coefficients). These findings are consistent with well-established associations in the literature whereby insufficient physical activity is related to overweight and obesity (Moschonis and Trakman, 2023), which in turn are associated with greater spinal loading and higher prevalence or severity of back pain (Ruiz-Fernandez et al., 2025). Prior studies suggest that low levels of physical activity may contribute to reduced muscular strength, diminished joint flexibility, and decreased spinal stability—factors that are plausibly linked to back problems (Kim and Yim, 2020; Al-Mudarres and Al-Shammari, 2024). Despite general consistency with these mechanisms, some studies have reported inconsistent findings (Bayartai et al., 2023). These discrepancies may reflect methodological heterogeneity (e.g., differences in activity measurement, population sampled, or clinical vs. non-clinical cohorts). For example, studies focused on chronic clinical populations may not generalize to broader student samples, whereas population-based studies capture a wider range of symptom severity.

In addition, physical inactivity was associated with LBP via BMI in our model: the indirect (mediated) path through BMI was small but statistically significant. While this sequential pattern aligns with the hypothesis that inactivity relates to higher BMI which in turn is associated with higher LBP scores (Jakicic et al., 2018; Jakicic et al., 2010), the cross-sectional data do not permit definitive conclusions about temporal ordering or causal mediation. Longitudinal data are needed to test whether inactivity leads to weight gain and subsequently increases LBP risk.

Within the SEM, BMI showed a significant direct path to LBP, indicating an association between higher BMI and greater LBP intensity. This observation aligns with systematic reviews reporting positive associations between higher BMI and LBP risk (Shiri et al., 2010) and with population studies from diverse settings (Siddiqui et al., 2022). Mechanistically, excess body weight—particularly abdominal adiposity—may alter spinal biomechanics and increase mechanical loading on lumbar structures; biological pathways such as chronic low-grade inflammation may also contribute to pain sensitization (Bayartai et al., 2023). Nevertheless, causal interpretation is not warranted on the basis of cross-sectional associations alone.

Another notable aspect of the model was that students who attended PE classes more regularly were less likely to be classified as inactive. This pattern suggests that structured exercise opportunities may help anchor activity routines within academic schedules; alternatively, attendance at PE may reflect broader lifestyle characteristics that co-occur with greater overall activity. While PE participation was associated with lower inactivity in our sample, this result does not establish that PE attendance is protective against LBP without further experimental or longitudinal evidence.

Gender and family income showed negligible associations with health anxiety in this sample, suggesting that psychological and behavioral variables may be more salient correlates of LBP in university environments than sociodemographic factors.

Several limitations should be acknowledged. The cross-sectional design prevents causal inference and limits conclusions about temporal sequencing. Reliance on self-report measures introduces potential reporting bias. Although the sample was large and drawn from multiple institutions across Guangdong Province, the findings may not generalize beyond Chinese undergraduates. Health anxiety was assessed using a brief screening instrument; future studies could use more comprehensive measures to distinguish health-specific worry from broader anxiety or distress.

Overall, the findings highlight meaningful associations linking psychological distress, health behaviors, and musculoskeletal symptoms within a university population, and they underscore the value of integrated analytic approaches (such as SEM) for examining multiple, concurrent pathways. Longitudinal and intervention studies are needed to determine whether modifying health anxiety, reducing sedentary behavior, or targeting weight management leads to improvements in LBP outcomes.

## 5 Conclusion

This cross-sectional SEM study investigated the associations among health anxiety, physical inactivity, participation in PE classes, and LBP in Chinese university students. The findings indicate that higher levels of health anxiety were associated with greater likelihood of physical inactivity, and that physical inactivity was associated with both higher LBP intensity and higher BMI. BMI also exhibited a direct association with LBP within the structural equation model. Participation in PE classes was inversely associated with physical inactivity.

These results point to a multifaceted pattern in which psychological characteristics, lifestyle behaviors, and physical health indicators co-occur among university students. Given the cross-sectional nature of the data, causal claims are not supported; instead, the study identifies empirical associations and potential pathways that warrant confirmation using longitudinal or experimental designs. Future research should examine temporal relationships and evaluate whether interventions targeting health anxiety, sedentary behavior, or weight management lead to reductions in LBP burden among young adults.

## Data Availability

The raw data supporting the conclusions of this article will be made available by the authors, without undue reservation.
